# Complete chloroplast genome and phylogenetic analysis of *Glebionis coronaria* (L.) Cass. ex Spach (Asteraceae)

**DOI:** 10.1080/23802359.2021.1966331

**Published:** 2021-08-18

**Authors:** Weiqiang Li, Shufang Jiang, Jie Wang, Youjian Yu, Zhujun Zhu

**Affiliations:** a Collaborative Innovation Center for Efficient and Green Production of Agriculture in Mountainous Areas of Zhejiang Province, College of Horticulture Science, Zhejiang A&F University, Hangzhou, Zhejiang, China; b Ningbo Academy of Agricultural Sciences, Ningbo, Zhejiang, China

**Keywords:** *Glebionis coronaria*, crown daisy, chloroplast genome, phylogenetic analysis

## Abstract

*Glebionis coronaria* (Asteraceae) is widely distributed in China, and it regulates the stomach, strengthens the spleen, reduces blood pressure, and reinforces the brain. In this study, the complete chloroplast genome sequence of *G. coronaria* was reported. The total chloroplast genome cycle was 149,750 bp, and it formed a large single-copy (LSC, 82,290 bp), a small single-copy (SSC, 18,414 bp), and two inverted repeats (IR, 24,523 bp) regions. The GC content of this genome was 36.35%. The whole-genome contained 128 genes, including 84 protein-coding genes, 36 tRNA genes, and eight rRNA genes. Phylogenetic analysis indicated that *G. coronaria* appeared within a clade comprised of *Chrysanthemum* species.

*Glebionis coronaria* (Asteraceae), which is native to Mediterranean regions, is annually propagated through seeds. Its stems can be up to 70 cm, while leaves are alternate with long, pinnate split, flowers yellow or white plus some wild *Chrysanthemum*-like features (Sulas et al. 1999; Basta et al. 2007). While its flowers are usually used for flower decoration in social and religious activities, they differ from those ordinary chrysanthemums in several manners (Teja et al. 2019). *Glebionis coronaria* has many beneficial effects, including anti-hypercholesterolemia, anti-fungal, anti-hyperglycemia, and anti-aging (Abd-Alla et al. 2014; Park et al. 2019). In the present study, the whole chloroplast genome of *G. coronaria* was characterized and assembled for the first time to reveal the genetic taxonomy at the molecular level and provide a basis for the evolutionary analysis of *G. coronaria* within the Aster family.

Fresh leaf samples of *G. coronaria* were acquired from the experimental farm of Zhejiang Agriculture and Forestry University, Hangzhou, Zhejiang Province, China (40.96°N, 117.44°E). The experiment was carried out following the standard protocol provided by Illumina (Illumina, San Diego, CA, USA). Total genomic DNA was extracted from fresh leaves via a modified CTAB procedure (Montero-Pau et al. 2018). We stored the genomic DNA in the College of Horticulture Science, Zhejiang Agriculture and Forestry University (ZAFUCC01). The DNA was segmented by mechanical interrupting (ultrasonic), and then the fragment size was selected by agarose gel electrophoresis. The sequencing library was formed by PCR amplification (NEBNext Ultra DNA Library Prep Kit for Illumina, San Diego, CA, USA) The constructed libraries were first inspected, and the libraries were sequenced using the Illumina NovaSeq platform. The SOAPdenovo2 v.2.04 software (Luo et al. 2012) was used to filter the raw sequencing data. The read length of the raw sequencing data was 150 bp, and approximately 3.9 Gb clean reads were obtained after moving adapters and unreliable reads by using Illumina NovaSeq (Illumina, San Diego, CA, USA). The filtered data were assembled by SPAdes version 3.10.1 (Bankevich et al. 2012). Then, the assembled genome was annotated by CPGAVAS2 version 2 (Shi et al. 2019).

The complete chloroplast genome of *G. coronaria* (GenBank accession number: MW874476) has a typical chloroplast genome circular structure with a length of 149,750 bp. This chloroplast genome has four typical subregions: one large single-copy (LSC, 82,290 bp) region, one small single-copy (SSC, 18,414 bp) region, and two inverted repeats (IR, 24,523 bp) regions. The average GC content of the whole chloroplast genome was 36.35%, and the corresponding values in the LSC, SSC, IR regions were 35.61%, 30.81%, and 43.09%, respectively. The whole-genome contained 128 genes, including 84 protein-coding genes, 36 tRNA genes, and eight rRNA genes.

Eleven complete chloroplast genomes from the *Chrysanthemum* species and two outgroups were downloaded from the GenBank database to investigate the phylogenetic position of *G. coronaria*. These sequences were aligned on Mafft version 7 software (Katoh and Standley 2013). The phylogenetic tree was constructed by the maximum likelihood method using Mega-X version 10.0.5 software with 1000 bootstrap replicates (Kumar et al. 2018). *C. coronarium* appeared within a clade comprised of *Chrysanthemum* species (Figure 1). Furthermore, it was found that *Cynara cardunculus* var. S*colymus* was in the same clade and was close to *C. coronarium*. This study provides a basis for resolving problems related to the phylogeny of *Chrysanthemum*, a genus with rapid speciation.

**Figure 1.**
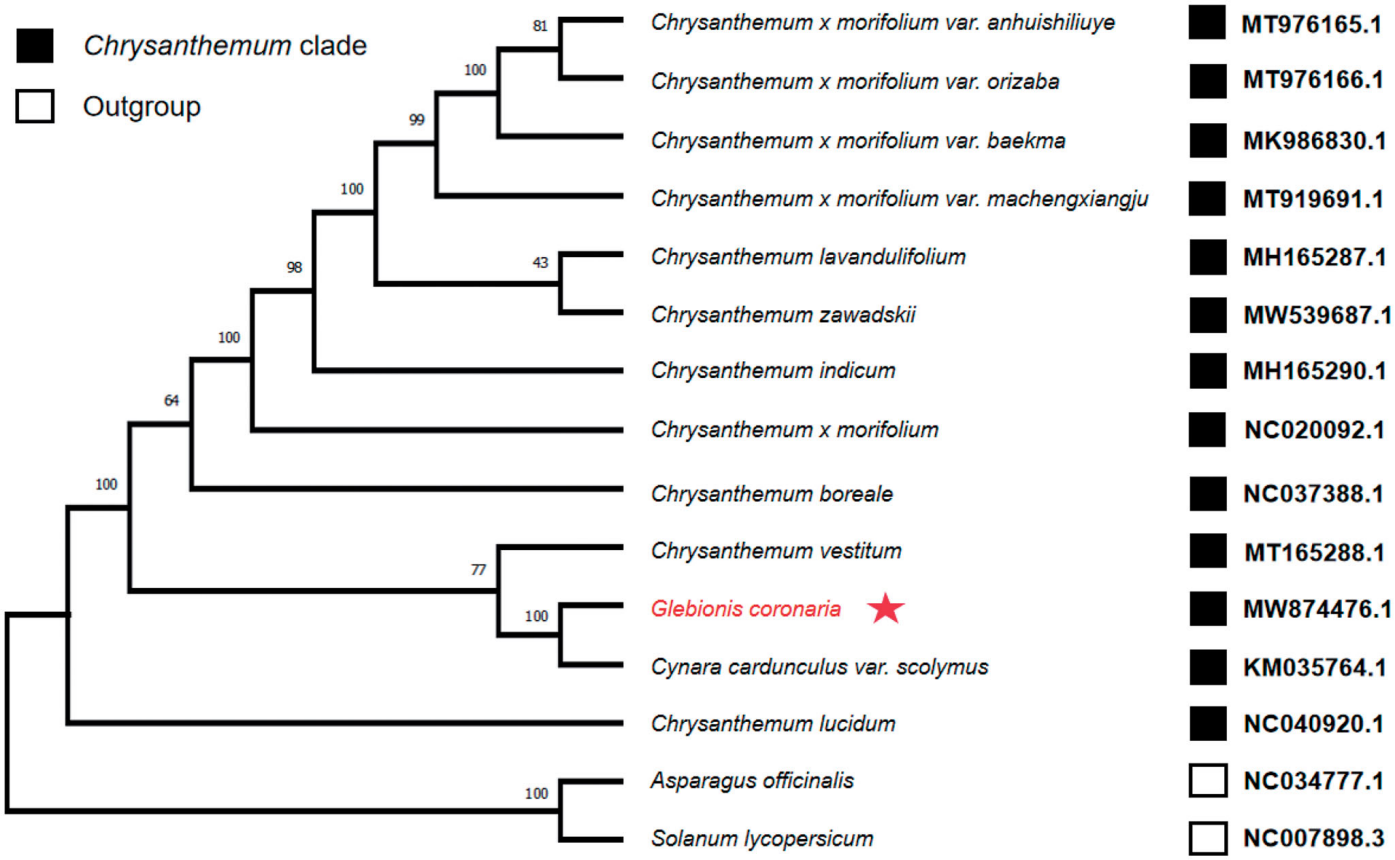
The maximum likelihood (ML) phylogenetic tree was founded on 15 selected complete chloroplast genomes. The bootstrap support values were shown above the branches. The position of *Glebionis coronaria* was marked with an asterisk and GenBank accession numbers were listed behind each species name.

## Data Availability

The data that support the findings of this study are openly available in GenBank of NCBI at https://www.ncbi.nlm.nih.gov, reference number MW874476. The raw sequencing data is deposited in GenBank (https://www.ncbi.nlm.nih.gov/) under BioProject ID PRJNA735919. The genomic DNA of *Glebionis coronaria* (Asteraceae) was stored in Collaborative Innovation Center for Efficient and Green Production of Agriculture in Mountainous Areas of Zhejiang Province, College of Horticulture Science, Zhejiang Agriculture and Forestry University. Youjian Yu is the administrator.
